# The Colonization of Synthetic Microbial Communities Carried by Bio-Organic Fertilizers in Continuous Cropping Soil for Potato Plants

**DOI:** 10.3390/microorganisms12112371

**Published:** 2024-11-20

**Authors:** Wenming Zhang, Shiqing Li, Pingliang Zhang, Xuyan Han, Yanhong Xing, Chenxu Yu

**Affiliations:** 1 College of Resources and Environmental Sciences, Gansu Agricultural University, Lanzhou 730070, China; sqli@ms.iswc.ac.cn (S.L.); 455069913@qq.com (X.H.); 2916268832@qq.com (Y.X.); 2 Dry land Agriculture Institute, Gansu Academy of Agricultural Sciences, Lanzhou 730070, China; zhangpl2007@163.com; 3 Department of Agriculture and Biosystem Engineering, Iowa State University, Ames, IW 50010, USA

**Keywords:** potato, synthetic microbial communities, bacterial community, fungal community, microbial function

## Abstract

Synthetic microbial communities (SynComs) play significant roles in soil health and sustainable agriculture. In this study, bacterial SynComs (SCBs) and fungal SynComs (SCFs) were constructed by selecting microbial species that could degrade the potato root exudates associated with continuous cropping obstacles. SCBs, SCFs, and SCB + SCF combinations were then inoculated into organic fertilizers (OFs, made from sheep manure) to produce three bio-organic fertilizers (BOFs), denoted by SBFs (BOFs of inoculated SCBs), SFFs (BOFs of inoculated SCFs), and SBFFs (BOFs of inoculated SCB + SCF combinations), respectively. The OF and three BOFs, with a chemical fertilizer (CK) as the control, were then used in pot experiments involving potato growth with soil from a 4-year continuous cropping field. Microbial diversity sequencing was used to investigate the colonization of SCBs and SCFs into the rhizosphere soil and the bulk soil, and their effects on soil microbial diversity were evaluated. Source Tracker analysis showed that SCBs increased bacterial colonization from the SBFs into the rhizosphere soil, but at a relatively low level of 1% of the total soil bacteria, while SCFs increased fungi colonization from the SFF into the bulk soil at a much higher level of 5–18% of the total soil fungi. In combination, SCB + SCF significantly increased fungi colonization from the SBFF into both the bulk soil and the rhizosphere soil. Overall, the soil fungi were more susceptible to the influence of the BOFs than the bacteria. In general, the application of BOFs did not significantly change the soil microbial alpha diversity. Correlation network analysis showed that key species of bacteria were stable in the soils of the different groups, especially in the rhizosphere soil, while the key species of fungi significantly changed among the different groups. LEfSe analysis showed that the application of BOFs activated some rare species, which were correlated with improvements in the function categories of the tolerance of stress, nitrogen fixation, and saprotroph functions. Mantel test analysis showed that the BOFs significantly affected soil physicochemical properties, influencing bacterial key species, and core bacteria, promoting potato growth. It was also noted that the presence of SynCom-inoculated BOFs may lead to a slight increase in plant pathogens, which needs to be considered in the optimization of SynCom applications to overcome continuous cropping obstacles in potato production.

## 1. Introduction

Microbiome engineering is being increasingly applied in the regulation of soil microbiomes and to improve crop productivity, in which the transplantation of microbial communities regulates the structure and function of soil microbial communities, mediates plant protection, and inhibits plant diseases [1]. Vaccination screening and the addition of efficient plant-growth-promoting rhizobacteria (PGPR) strains to improve soil microbial communities have become common methods used to increase crop yield [2]. However, the utilization of exogenous microbial strains in these approaches has faced difficult challenges because the survival and functional maintenance of these strains as they are transplanted into soil depend on communication and interaction between them and the indigenous microorganisms in the soil [3]. The synthetic microbial community (SynCom) approach is an emerging technique that involves co-culturing multiple taxa, under well-defined conditions, to mimic the structure and function of a microbiome. SynComs can better adapt to soil surroundings than individual strains due to the division of tasks among its member strains and the promotion of collaboration among member strains to aid in biosynthesis [4].

The effects of SynComs on anti-plant pathogens [5], stressors [6] and soil health [7] have been investigated. However, the construction of SynComs for specific applications is still a very tough task. Top-down and bottom-up approaches have both been explored [8]. Top-down methods focus on the design of the function and purpose of SynComs to achieve certain goals [9], while bottom-up methods use plant–bacterium binary association assays to define the groups of bacteria that have similar effects on the host plant and then construct partially overlapping bacterial communities that optimize their functions and interactions with host plants [10]. Regardless of the SynCom construction methods used, identifying and isolating efficient and multifunctional microbial strains are the keys to SynCom construction. The colonization and action of the SynCom consortia isolated from different environments on soil and host plants could be completely different depending on the soil environment as well as the interactions between microorganisms and plant roots [7]. In addition, selected SynCom consortia often need carriers to support the survival and reproduction of member strains. Several studies have proven that organic fertilizer provides effective carrier systems for SynComs that can promote microbial soil colonization. The resultant bio-organic fertilizer (BOF) could increase soil microbial functionality [11,12]. 

Furthermore, studies have also shown that plant root secretions have significant effects on the soil’s microbial biodiversity [13,14]. For example, previous studies have confirmed that during the continuous cropping (CC) of potato plants, the root exudates of the potato plants drive the formation of rhizosphere microflora [15]. As the years of CC increased, the accumulation of autotoxins from the root exudate of the potato plants leads to changes in the rhizosphere as well as the bulk soil microbial community and tends to have increasingly adverse effects on the plant growth and yield [15]. A possible approach to negate some of these adversities facing potato plants during CC is to colonize the soil with SynComs of enriched microbial strains that can degrade autotoxins as well as other allelochemicals secreted by the potato plants during CC. As of today, research on such approaches is still quite limited. 

Therefore, this study aimed to investigate how the SynComs (i.e., constructs of microbial strains capable of degrading potato root exudates) carried by BOFs could colonize into the soil of CC pots, and what their impacts were on the soil microbial communities and biodiversity. More specifically, bacterial SynComs and fungal SynComs were constructed by selecting strains that could degrade potato root exudates isolated from the soil of CC fields of potato, and sheep manure compost was used as the carrier systemby which the SynComs would make bio-organic fertilizers (BOFs). To study the effects and interactions between the BOFs, potato plants, and soil, the BOFs were utilized in pot experiments of potato growth, with soils collected from potato fields of 4-year CC. Rhizosphere and bulk soils from each pot were then collected and sequenced along with SynCom constructs and BOF samples to characterize the soil colonization of the SynCom strains from the BOFs, and changes in the bacterial and fungal biodiversity and communities in the soil were assessed. The findings of this study provide foundational information for the construction of effective SynComs and the development of specialized BOFs that can potentially negate the continuous cropping obstacle faced during potato plantation, which could bring significant benefit to potato farmers.

## 2. Materials and Methods

### 2.1. SynCom Construction

In total, 75 fungi and 115 bacteria strains, which could degrade root exudates of potato under continuous cropping (CC), were screened from soils of potato fields under annual rotation using potato root exudates. These were collected from potato fields of 4 and 7 years of CC, used as the sole carbon and nitrogen sources, and were then propagated on agar plates for 10 generations and preserved. The 115 bacteria and 75 fungi strains propagated were inoculated into LB (Luria–Bertani) and SDB (liquid sabouraud medium) broth, and cultivated at 28 °C under constant shaking (150 rpm) for 72 h to form bacterial SynCom (SCB) and fungal SynCom (SCF) constructs, respectively. SCBs and SCFs were sequenced to determine the composition of their consortium, and it was confirmed that the SCBs had 76 species, while the SCFs had 85 species. The results are shown in Tables S1 and S2.

### 2.2. Preparation of BOFs

SCB and SCF constructs were inoculated into LB (Luria–Bertani) and SDB (liquid sabourand medium) broth, and cultivated at 28 °C under constant shaking (150 rpm) for 72 h (with an OD600 value greater than 0.5, the total microbial concentration reached 10^9^ CFU/L). Then, the cultures were added to the mature organic fertilizer (sheep manure) at a rate of 10% (*v*/*w*) to make various BOF samples; cultured SCBs and SCFs were mixed to form SCB + SCF at a 1:1 *v*/*v* ratio. A mix made of equal amounts of non-inoculated liquid culture/sheep mature was used as the blank (OF). The liquid cultures (both the sample and control) and the mature were mixed thoroughly, and they were then fermented for one week to form the BOFs. Three kinds of BOF were formed, and denoted by SBF (inoculated with SCBs), SFF (inoculated with SCFs) and SBFF (inoculated with SCBs and SCFs at 1:1), respectively. Samples of OF, SBF, SFF and SBFF were collected and frozen at −80 °C for sequencing.

### 2.3. Design of Pot Experiments of Potato Growth

Topsoil (0–20 cm) from a 4 yr continuous cropping potato field was collected in 2023 from the National Soil Quality Stability Observation and Experiment Station of Gansu Academy of Agricultural Sciences, Gansu Province, Northwestern China (104°36′ E, 35°35ʹ N). The averages of altitude, annual temperature, annual precipitation, annual evaporation, and the frost-free period of the site were 1970 m, 6.2 °C, 415 mm, 1531 mm, and 146–149 day/year, respectively. The physical–chemical properties of the soil were as follows: total organic carbon 9.04 g·kg^−1^, total nitrogen 0.78 g·kg^−1^, available phosphorus 8.25 mg·kg^−1^, available potassium 83.67 mg·kg^−1^, and pH 8.30. 

Soil was sieved through a 10.0 mm sieve after air-drying. Each pot was packed with 2.0 kg of soil; chemical fertilizers were applied at 0.1 gN/kg soil, N/P = 2:1 (urea for N, and potassium dihydrogen phosphate for P). Then, 5 groups were set up with different fertilizers (OF, SBF, SFF and SBFF), applied at 4.5 g·pot^−1^ (converted at 5250 kg·hm^−2^), which were denoted as F, FSB, FSF and FSBF, respectively. There was a control group (CK) to which no organic fertilizer was added. Then, 6 replications were conducted for each group. Fertilizers were mixed with soil fully. One plant (Lushu no.3) was seeded 4 cm from the surface in each pot. Pots were placed in the greenhouse on the campus of Gansu Agricultural University from 5 May–5 July 2023.

### 2.4. Samples Collection

Plants were dug out at the blossoming stage, and paired bulk soils (B) and rhizosphere soils (R) were obtained using the “soil adhering to fine roots after shaking” method [16]. In brief, soil was obtained by shaking the roots gently until no more large soil aggregates, defined as bulk soil, were dropped; then, soil adhering to the fine roots was brushed down with sterile brushes, which was defined as rhizosphere soil. Soil samples were split into three parts: one part was kept fresh for pH and EC determination, one part was air-dried for the determination of soil physicochemical properties, and another was frozen at −80 °C for sequencing. The soil samples from three pots (randomly selected), along with SynCom samples (SCFs and SCBs) as well as BOF samples, were sent to Biomarker Technologies Co., Ltd. (Beijing, China), for gene sequencing.

### 2.5. Plant Growth and Soil Physicochemical Properties Determination

Plant height and diameter (at the soil surface) were measured with a ruler and vernier caliper, respectively. Dry plant mass was determined as follows: the plant above the ground was collected, blanched in an oven at 105 °C for half an hour, and then dried at 70 °C and weighed. Soil pH and EC were determined using a pH meter and an electrode, respectively [17]. The total nitrogen (TN) of the soil was determined by the Kjeldahl method [18]. The available potassium (AK) of the soil was extracted with ammonium acetate and was determined by the flame photometric method, the available phosphorus (AP) of the soil was extracted with 0.5 M NaHCO_3_ and determined by the molybdenum–antimony resistance colorimetry method [17], and soil organic carbon (TOC) was determined by heating and titrating with K_2_Cr_2_O_7_ and H_2_SO_4_ [17].

### 2.6. DNA Extraction and Sequencing

The total genomic DNA of each sample (SynCom samples (SCFs and SCBs), BOF samples, and soil samples) was extracted with the TGuide S96 Magnetic Soil/Stool DNA Kit (Tiangen Biotech (Beijing) Co., Ltd. (Beijing, China)) according to the manufacturer’s instructions, and the DNA concentration was measured with the Qubit dsDNA HS Assay Kit and Qubit 4.0 Fluorometer (Invitrogen, Thermo Fisher Scientific, Hillsboro, OR, USA). Fungal ITS region was amplified using the primer pair ITS1F (5’-CTTGGTCATTTAGAGGAAGTAA-3′) and ITS2 (5’-GCTGCGTTCTTCATCGATGC-3′), and the thermal cycling conditions were 95 °C for 5 min, 15 cycles of 95 °C for 1 min, 50 °C for 1 min, and 72 °C for 1 min, followed by 72 °C for 7 min [15]. The bacterial 16S rRNA gene V3–V4 region of 16S rRNA was amplified using the primer pair 338 F (5’-ACTCCTACGGGAGGCAGCA-3′) and 806 R (5’-GGACTACHVGGGTWTCTAAT-3′), and the thermal cycling conditions were pre-denaturation at 98 °C for 2 min, denaturation at 98 °C for 15 s, annealing at 55 °C for 30 s, extension at 72 °C for 30 s, and final extension at 72 °C for 5 min (30 cycles) [15]. The PCR amplicons were gel-purified with Agencourt AMPure XP Beads (Beckman Coulter, Indianapolis, IN, USA). The resultant PCR products were combined at equimolar concentrations and we obtained Illumina NovaSeq 6000 (Illumina, Santiago, CA, USA) for sequencing (250 × 250 bp) from Beijing Biomarker Technologies Co., Ltd. (Beijing, China). Raw reads were filtered with Trimmomatic (version 0.33); they were then identified. We removed primer sequences with cutadapt (version 1.9.1) to obtain clean reads, and then denoised the results with QIIME2 (version 2020.6) to obtain non-chimeric reads.

### 2.7. Data Processing and Analysis

Operational taxonomic units (OTUs) were clustered with 97% similarity using Naive Bayesian classifiers of QIIME2 in the SILVA database with a confidence threshold of 70%. Source Tracker analysis was used to identify the colonization of SynCom strains into BOFs and soil with R (Source Tracker package, version 3.1.1). Shannon index values were calculated and displayed by the QIIME2 and mothur (ggplot2 package, version 3.1.1; ggpubr, v1.22.2). Principal co-ordinate analysis (PCoA) was used to identify the difference between samples with R (ggplot2 package, version 3.1.1). Bugbase phenotypic prediction was used to classify the microbial phenotype with bugbase (version 0.1.0); FAPROTAX function prediction was used to predict bacterial community function with FAPROTA (version 1.2.6); Fungi Functional Guild (FUNGuild) was used to predict fungal community function. Correlation network analysis was used to identify the relationship between species and find key species using R (psych package, version 3.6.1). Line discriminant analysis (LDA) effect size (LEfSe) was used to identify biomarkers with statistical difference using python2. The Mantel test was used to identify the correlation between microbial community and environmental factors using R (vegan, ggplot2, ggcor; version 3.1.1). A statistical diagram was plotted through the CNSknowall site (https://cnsknowall.com/index.html#/HomePage, accessed on 21 October 2024).

## 3. Results

### 3.1. Colonization of SynCom Strains into the BOFs and the Soil

**Figure 1:**
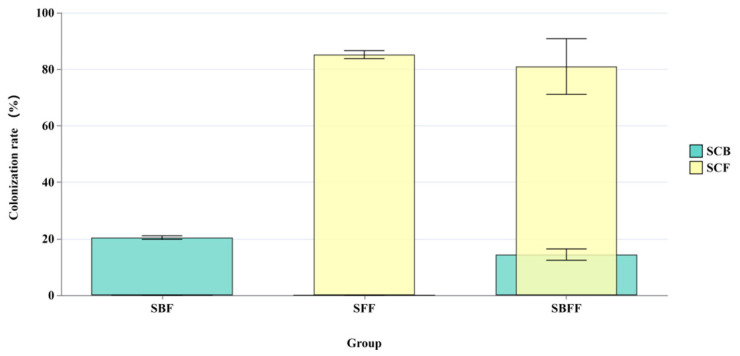
Source Tracker analysis of SynCom colonization into the BOF (SBF: BOF inoculated with SCBs; SFF: BOF inoculated with SCFs; SBFF: BOF inoculated with SCB + SCF).

Source Tracker analysis could reflect the SynCom colonization state. SCBs colonized into the BOF (i.e., SBF, SBFF) at about 14.4–20.4% of the total bacteria count, while the value for SCFs was about 81–85.2% of the total fungi count (Figure 1), indicating that SCF strains colonized the BOF much easier and more extensively than the SCB strains. In addition, with the presence of both SCBs and SCFs (i.e., the SBFF samples), the colonization of the BOF by either fungi or bacteria was decreased compared to the case when either SCBs or SCFs were used alone.

**Figure 2:**
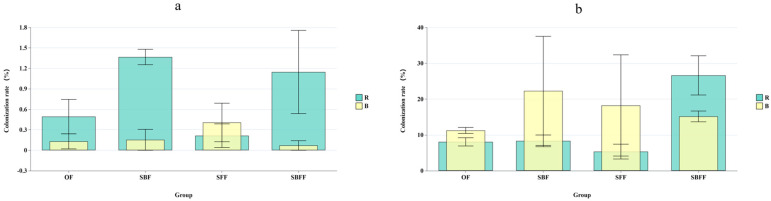
Source Tracker analysis of microbial colonization from BOF into bulk soil (B) and rhizosphere soil (R): (**a**). soil bacteria; (**b**). soil fungi (OF: organic fertilize; SBF: BOF inoculated with SCBs; SFF: BOF inoculated with SCFs; SBFF: BOF inoculated with SCB + SCF).

Subsequently, the colonization of SynComs, carried by BOFs, into the bulk (B) and rhizosphere (R) soil was also assessed using Source Tracker analysis. With SCBs and SCB + SCF-inoculated BOFs (i.e., SBF and SBFF), the colonization of bacterial strains from the BOFs into the rhizosphere (R) soil was increased, but was still at low levels (from 0.49% in F to 1.37% in FSB group and 1.15% in FSBF group), while the SCF-inoculated BOF (SFF) led to a slight drop in bacterial colonization into the rhizosphere soil (0.49% to 0.21%). Meanwhile, the colonization of bacterial strains into the bulk soil did not seem to be affected by the SynComs coming from the BOF (Figure 2a). 

A different, yet intriguing scenario was observed with SCF-inoculated BOFs (i.e., SFFs and SBFFs). As shown in Figure 2b, fungi colonization from BOFs into bulk soil was increased by all BOFs. Intriguingly, the use of SCB-inoculated BOFs (SBFs) led to greater colonization of fungi (22.3%) than that of SCFs (18.22%)- and SCB + SCF (15.15%)-inoculated BOFs. Yet, the use of SCBs or SCFs alone did not change the colonization rate into rhizosphere soil (R), but the combined SCB + SCF-inoculated BOF (SBFF) increased the colonization of fungi into the rhizosphere soil significantly (8.05% to 26.62%). Overall, fungal colonization was significantly greater than bacterial colonization, indicating that soil fungi were easier to regulate than bacteria. However, the complex patterns observed for the fungal colonization with different BOFs suggested that the colonization of fungi into the rhizosphere and bulk soil was not a simple process, and more study was needed to understand it better.

Examining the abundance of bacteria in SCBs, the top 10 species were *Pseudomonas*, *Enterobacter*, *Acinetobacter*, *unclassified*_*Enterobacteriaceae*, *Brevundimonas*, *Bacillus*, *Lysinibacillus*, *Paenibacillus*, and *unclassified*_*Muribaculaceae* (S1). The relative abundance values of *Pseudomonas* and *Enterobacter* were 43.8% and 35.6%, respectively. *Pseudomonas*, *Enterobacter*, *Acinetobacter*, *Brevundimonas*, *Bacillus*, and *Paenibacillus* already existed in the non-inoculated organic fertilizer as well as the BOFs (S3), while the inoculation of SCBs did increase the relative abundance of most bacteria, except for *Bacillus*. Meanwhile, only *Pseudomonas*, *Brevundimonas*, and *Bacillus* existed in soil at over 0.1% levels. After the addition of the BOFs, the presence of *Pseudomonas* and *Brevundimonas* increased in soil, while the levels *Bacillus* remained unchanged (S4). *Pseudomonas*, *Enterobacter*, and *Bacillus* were most frequently used in SynComs according to the literature [19]. 

The top 10 fungi in terms of SCFs were *Candida*, *Aspergillus*, *Cladosporium*, *unclassified*_*Ascomycota*, *unclassified*_*Saccharomycetales*, *Mortierella*, *Fusarium*, *Galactomyces*, *unclassified*_*Agaricales* and *unclassified*_*Basidiomycota* (S5), all of which existed in the non-inoculated organic fertilizer and the BOFs, indicating these species existed widely in organic fertilizers. The relative abundance of *Fusarium*, *Galactomyces*, unclassified_*Agaricales*, and *unclassified*_*Basidiomycota* increased with the inoculation of SCF, while that of Candida decreased (S6). It is interesting that the relative abundance of other fungal species increased upon the application of SCB-inoculated BOF, yet decreased upon the application of SCF-inoculated BOF, except for *unclassified*_*Ascomycota*, indicating that complex interactions between bacteria and fungi played critical roles in microbial survival and reproduction. *Unclassified*_*Ascomycota*, *Plectosphaerella* and *Chalara* were among the top 3 most abundant bacteria found in soil (S6). Upon the application of the BOFs, the presence of *Mortierella* increased in bulk soil while it decreased in rhizosphere soil; *unclassified*_*Ascomycota* was the opposite, decreasing in bulk soil but increasing in rhizosphere soil. The presence of *Candida* increased in both the rhizosphere soil and bulk soil of F and FSF groups, the presence of *Fusarium* increased in the bulk soil of F and the rhizosphere soil of the FSF group, and presence of *Galactomyces* increased in both the bulk soil and the rhizosphere soil of F. It was noticeable that *Pleuroascus*, *Coprinus*, *Aaosphaeria*, *Coprinellus*, and *unclassified*_*Mycosphaerellaceae* only existed in the rhizosphere soil of the various samples, suggesting that these fungi were more closely tied to the plants.

### 3.2. Effects of SynCom/BOF on Soil Microbial Diversity 

**Figure 3:**
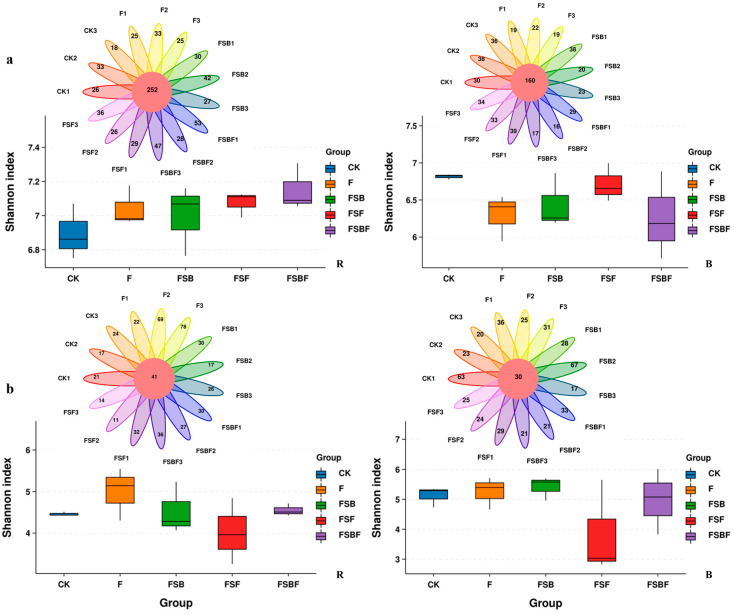
Effect of application of BOFs on soil microbial alpha diversity and species composition (B: bulk soil; R: rhizosphere soil: (**a**), bacteria, (**b**): fungi). CK: no organic fertilizers applied; F: organic fertilizer applied; FSB: SBF applied; FSF: SFF applied; FSBF: SBFF applied.

The application of BOFs did not seem to change soil microbial alpha diversity much (Figure 3). Across the groups, the Shannon index of bacteria in the rhizosphere soil slightly increased, while the Shannon index of bacteria in the bulk soil slightly decreased; however, the changes appear not to be significant. The Shannon indexes of fungi decreased in the FSF group, while they slightly increased in the F group. A Veen plot of species composition showed that the application of BOFs changed microbial community. Some 252 and 160 bacteria (core bacteria) commonly existed in the rhizosphere soil and the bulk soil of all groups. BOFs appeared to increase specific bacteria in the rhizosphere soil, while the decreased specific bacteria in the bulk soil. OF decreased specific bacteria in both rhizosphere soil and bulk soil.

Overall, 41 and 30 fungi (core fungi) were observed to exist in the rhizosphere soil and the bulk soil of all groups. Similar to the case of bacteria, the application of OF and BOFs led to increases in specific fungi in the rhizosphere soil, except for FSF, and decrease in specific fungi in the bulk soil, except for FSB.

**Figure 4:**
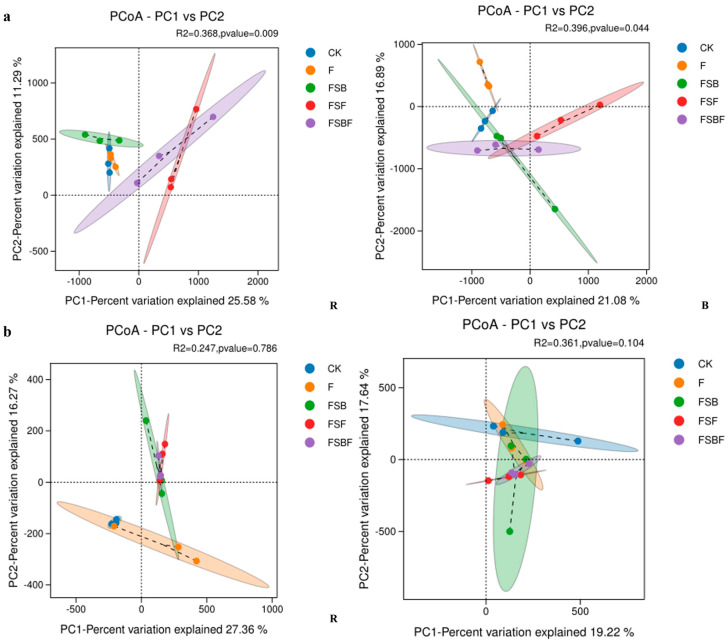
Effect of bio-organic fertilizer implication on soil microbial beta diversity (B: bulk soil; R: rhizosphere soil: (**a**): bacteria, (**b**): fungi). Ellipses represent a 95% confidence interval. CK: no organic fertilizers applied; F: organic fertilizer applied; FSB: SBF applied; FSF: SFF applied; FSBF: SBFF applied.

Notably, the application of BOFs significantly changed soil microbial beta diversity (Figure 4). PCoA analysis showed that BOFs significantly affected bacterial communities in both rhizosphere soil and bulk soil, while they did not significantly affect fungal communities. FSF and FSBF groups were clearly separated from the others; these observations confirmed the earlier observations made with the Source Tracker analysis. 

### 3.3. Effect of Application of BOFs on Status of Soil Microbial Communities 

**Figure 5:**
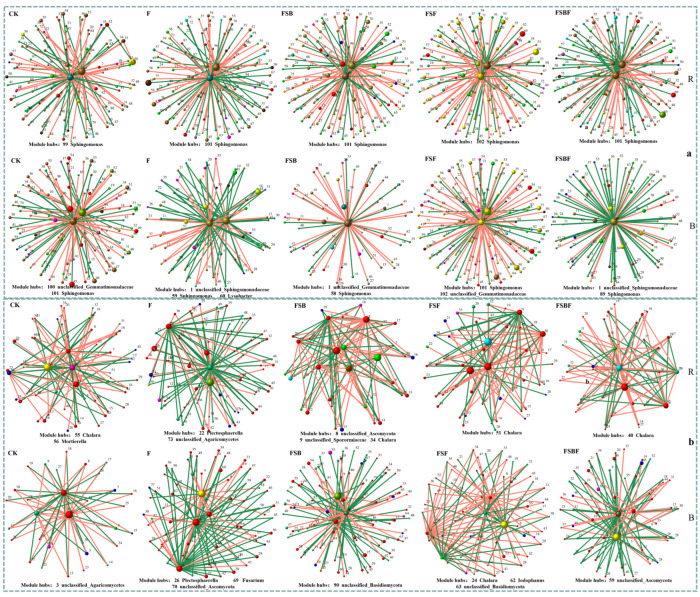
The correlation network diagram of each group (B: bulk soil; R: rhizosphere soil: (**a**), bacteria, (**b**): fungi). The correlation network was constructed as Spearman’s correlation, with a genera relative abundance of >0.1%, r > 0.8, and *p* < 0.05. Red lines represent significant positive relationships, and green lines denote negative relationships. The genera shown on these figures are the module hub (key species) in each network diagram (ZI (within-module connectivity), with >2.5 and PI (among-module connectivity) < 0.62. CK: no organic fertilizers applied; F: organic fertilizer applied; FSB: SBF applied; FSF: SFF applied; FSBF: SBFF applied.

The correlation network could reflect relationships between microorganisms, which could be used to identify module hubs; these were the key species according to Xing et al. [15]. As shown in Figure 5, the correlation network showed that key bacterial species were stable among different groups, especially in the rhizosphere soil, while key fungal species significantly changed among different groups. *Sphingomonas* appeared to be the key bacterial species in the rhizosphere soil and bulk soil for all groups. In addition, *unclassified_Gemmatimonadaceae* was a key bacterial species in the bulk soil of the control (CK), the FSB, and the FSF groups; *unclassified_Sphingomonadaceae* was a key bacterial species in the bulk soil of F and FSBF group; and *Lysobacter* was a key bacterial species in the bulk soil of F alone. 

As to the fungi, *Chalara* was a key fungal species in the rhizosphere soil of all groups, except for F, while it was only a key fungal species in the bulk soil of the FSF group. *Mortierella* in CK, *Plectosphaerella* and *unclassified_agaricomycetes* in F, *unclassified_ascomycota* and *unclassified_sporormiaceae* in FSB were all key fungal species in the rhizosphere soil. Key fungal species in the bulk soil of different groups were quietly different, and more similarity was observed between the pair of F and FSBF groups, and the pair of FSB and FSF groups. In addition, *unclassified_ascomycota* was a key fungal species in the bulk soil of F and FSBF groups; *unclassified_basidiomycota* was a key fungal key species in the bulk soil of FSB and FSF groups. 

The statistics of correlation network diagram (Figure 5) also showed that FSF significantly increased the positive bacterial correlation in both the rhizosphere soil and the bulk soil. FSBF significantly decreased the positive bacterial correlation in the rhizosphere soil, and FSB significantly increased it. As for fungi, FSF and FSBF significantly increased the fungi positive correlation in the rhizosphere soil, while FSB and FSBF significantly decreased fungi positive correlation in the bulk soil. 

These results showed that the application of the SynCom-inoculated BOFs changed correlations between microorganisms, thereby altering community structures and functions. Notably, the bacterial community was more stable and more difficult to regulate than the fungal community; and the microbial community in the rhizosphere soil was more stable than that of the bulk soil.

**Figure 6:**
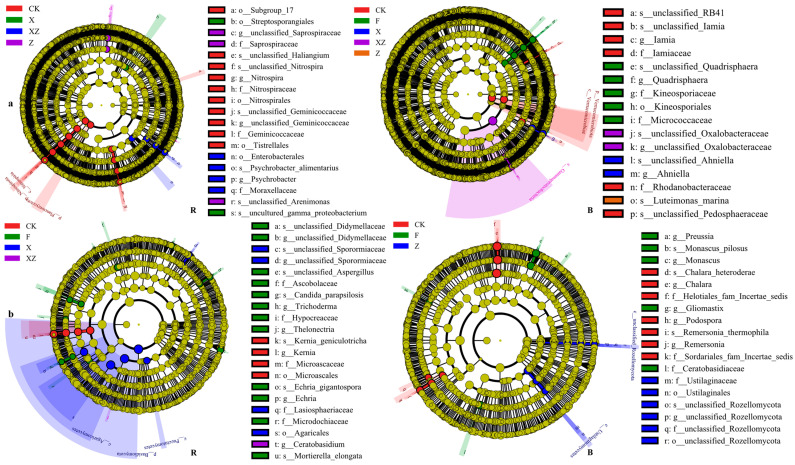
Line discriminant analysis (LDA) effect size (LEfSe) analysis (B: bulk soil; R: rhizosphere soil; (**a**), bacteria, (**b**): fungi). CK: no organic fertilizers applied; F: organic fertilizer applied; FSB: SBF applied; FSF: SFF applied; FSBF: SBFF applied. The figures show the genera with LDA scores greater than 3.0.

LEfSe analysis can be used to identify biomarkers associated with different groups. Bacterial LEfSe analysis showed that thirteen bacteria were the biomarkers for the control group (CK) in the rhizosphere soil, and eight bacteria were the biomarkers for the control group in the bulk soil. The application of BOFs led to the appearance of different biomarkers, such as *unclassified_Saprospiraceae*, *f_Saprospiraceae*, and *s_unclassified_Arenimonas* in the FSF group of the rhizosphere soil; *f_Moraxellaceae*, *g_Psychrobacter*, *o_Enterobacterales*, and *s_Psychrobacter-alimentarius* in the FSBF group of the rhizosphere soil; *o_Streptosporangiales* and *s_uncultured_gamma_Proteobacterium* in the FSB group of the rhizosphere soil; *s_Luteimonas_marina* in the FSF group of the bulk soil; *c_Gammaproteobacteria*, *s_unclassified_Oxalobacteraceae*, and *g_unclassified_Oxalobacteraceae* in FSBF group of the bulk soil; and s_*unclassified_Ahniella* and *g_Ahniella* in the FSB group of the bulk soil. The abundances of these bacteria were mostly below 0.1%, as shown in Figure 6a. 

Fungal LEfSe analysis showed that four fungi were biomarkers for the control (CK) in the rhizosphere soil, and seven fungi were biomarkers for the control (CK) in the bulk soil. As in the case of bacterial biomarkers, the application of various BOFs brought new fungal biomarkers, such as *g_ Ceratobasidium* in the FSBF group of the rhizosphere soil; *p_Basidiomycota*, *c_Agaricomycetes*, *o_Agaricales*, *s_unclassified-Sporormiaceae*, *g_unclassified-Sporormiaceae*, *f_Lasiosphaeriaceae*, and *c_Pucciniomycetes* in the FSB group of the rhizosphere soil; and *s_unclassified_Rozellomycota*, *f_unclassified_Rozellomycota*, *g_unclassified_Rozellomycota*, *c_unclassified_Rozellomycota*, *o_unclassified_Rozellomycota*, *c_Ustilaginomycetes*, and *f_Ustilaginales* in the FSF group of the bulk soil. The abundances of these fungi were also mostly below 0.1%, as shown in Figure 6b. Apparently, the application of BOFs mainly affected rare microorganisms in the soil. 

**Figure 7:**
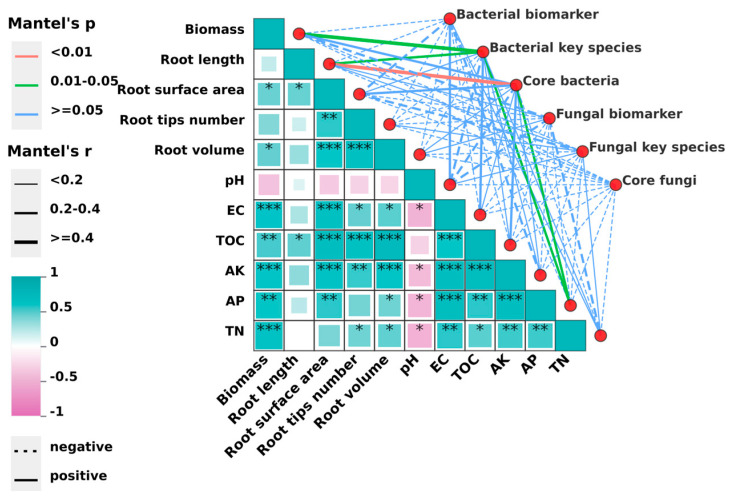
Mantel test analysis of plant growth, environmental factors, and microbial community (biomarkers were analyzed according to Figure 6, key species were analyzed according to Figure 5, and the fraction of the core bacterial/fungal species analyzed with a Veen plot are shown in Figure 3). *, **, and *** represent *p* < 0.05, *p* < 0.01, *p* < 0.001, respectively; a dark green square represents significant positive relationships, and a pink square represents negative relationships. The darker the color or the larger the square area, the greater the absolute value of the correlation coefficient; red lines represent *p* < 0.01, green lines represent 0.01 < *p* < 0.05, blue lines represent *p* ≥  0.05, — represents a positive relationship, --- represents a negative relationship, and the width of the line represents the magnitude of the correlation coefficient.

Mantel test analysis showed that soil EC, TOC, AK, AP, and TN were significantly correlated with root morphology and plant growth (Figure 7). The key bacterial species and core bacteria were significantly positively correlated with AP, while bacterial biomarkers, fungal biomarkers, fungal key species, and core fungi were not significantly correlated with environmental factors, indicating that they might be influenced by interactions between microorganisms. Additionally, bacterial key species and core bacteria were significantly positively correlated with root length, and bacterial key species was significantly positively correlated with biomass. However, fungal biomarkers, fungal key species, and core fungi were negatively correlated with nearly all plant indicators. These results indicated that BOFs significantly affected soil physicochemical properties, which influenced bacterial key species and core bacteria, and possessed physicochemical properties that could potentially promote potato growth.

### 3.4. Effects of Application of BOFs on Soil Microbial Function

**Figure 8:**
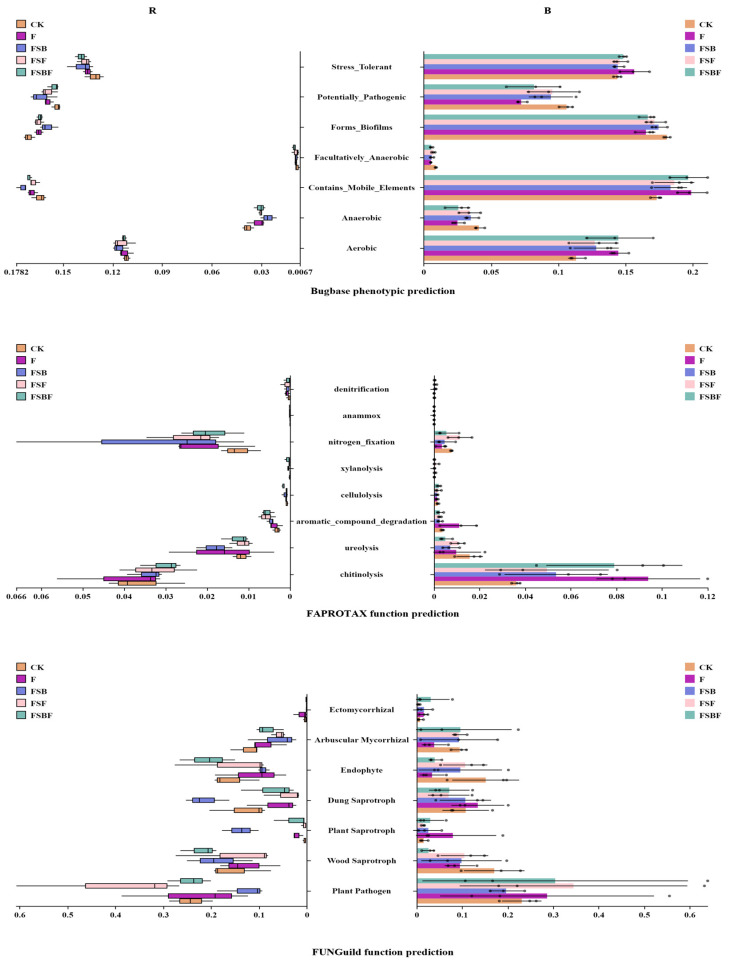
Function prediction of microbial community (R: rhizosphere soil; B: bulk soil). CK: no organic fertilizers applied; F: organic fertilizer applied; FSB: SBF applied; FSF: SFF applied; FSBF: SBFF applied.

Function prediction, as shown in Figure 8, suggested that the application of BOFs also changed the functions of the microbial communities. Bugbase phenotypic prediction of the bacterial community showed that application of BOFs improved stress tolerance in the rhizosphere soil, but decreased the stress tolerance function of microbes in the bulk soil. In addition, data suggested that the application of BOFs may lead to increases in the pathogenicity of the rhizosphere soil but decreases in the bulk soil. The FAPROTAX function prediction of bacteria community showed that functions associated with cellulolysis, xylanolyysis, and aromatic_compound_degradation in the rhizosphere soil were improved by BOFs, while, functions associated with nitrogen_fixation, anammox and denitrification were also improved in the rhizosphere soil.

FUNGuild function prediction showed that FSB and FSBF significantly improved functions associated with plant saprotroph and wood saprotroph in the rhizosphere soil, while it suppressed Arbuscular Mycorrhizal fungi (AMF) activity in the rhizosphere soil and limited wood saprotroph and endophyte activities in the bulk soil. Meanwhile, FSBF was predicted to improve endophyte activities in the rhizosphere soil, and FSB was predicted to increase the presence of dung saprotroph in the rhizosphere soil. In addition, FSF was predicted to increase plant pathogens in both the rhizosphere soil and the bulk soil, while FSB was predicted to have the exact opposite effect in the plant pathogens. 

## 4. Discussion

SynCom colonization into the soil as well as interactions between these add-on microbes and plants can affect the soil microbial community and diversity, which determined the overall functionality of the soil microbiota [20]. Global soil ecosystem data also indicated that soil biodiversity drives soil ecosystem functions [21]. Hence, knowledge on SynCom construction and colonization is of critical importance in driving their proper applications. 

### 4.1. SynCom Colonization from BOFs into Soil and Its Impact on Soil Microbial Diversity

SynComs could resist environmental disturbance and the invasion of other species into the soil better than alternatives, improving soil fertility and health [22]. Numerous studies were conducted on SynCom construction theories, and large amounts of microbial strains have been screened [23]. However, on average, the currently used SynComs typically have low complexity (<20 strains), which limits their ability to simulate the biological properties of natural microbial communities [22]. Furthermore, the soil adaptability of SynComs designed and developed in the culture medium needs to be tested, and how well SynComs can colonize into the soil is crucial for achieving its functions. Many studies have confirmed the role of SynComs in improving soil microbial diversity and the productivity of plants [24], but relatively little attention has been paid to the rate of colonization of SynComs into soil, which should be an important factor to be considered in the design of SynComs. For example, SynCom abundance was shown to drop significantly 2 weeks after inoculation [25], indicating the colonization of microorganisms in soil was limited. SynCom colonization in natural environment is a tough task, due to its interaction with soil native microbes and host plants. Hence, maintaining the stability and timeliness of SynCom colonization is a challenge for the proper application of SynComs [20]. The application of organic fertilizer also has impact on soil microbiota. Organic fertilizer was shown to activate rare microbes in the soil, but the effects were short-lived. After 2 weeks of application, 78% of genera related to organic fertilizer were not detected again [26]. The combined effects of SynComs and organic fertilizer are hence worthy of investigation. This study found that bacterial SynComs were less effective than fungal SynComs in their ability to colonize in both organic fertilizer and soil; this may be owing to the much higher counts of bacteria in nature [27], which induce more severe competition for the SybCom. This was also supported by the results of sequencing: bacterial counts were 2 and 6 times higher than fungal counts in compost and soil, respectively. It was also shown that, for potato, bacterial SynCom-inoculated BOFs significantly increased bacterial colonization in the rhizosphere soil, while fungal SynCom-inoculated BOFs significantly increased fungal colonization in the bulk soil. In addition, bacterial SynCom-inoculated BOFs also increased fungal colonization in the bulk soil, but fungal SynCom-inoculated BOFs could not increase bacterial colonization. The combined use of bacterial/fungal SynComs was shown to be effective in increasing fungal colonization in both the rhizosphere soil and the bulk soil. 

Microbial diversity was crucial to soil health and functionality [28]. Methods involving inoculating microorganisms and organic fertilizers are commonly used to improve the soil microbial community’s structure and diversity [20]. Some research found that the application of SynComs could increase bacterial alpha diversity and reshaped microbial communities in soil [7], but others also reported cases where the application of SynComs led to decrease in soil microbial diversity [29]. Hence, factors affecting soil microbial diversity are very complex, and are deeply affected by soil characteristics and interactions between plant roots and microbes. To effectively use SynCom/BOF systems to improve soil biodiversity, they must be carefully designed, constructed, and applied. The SynCom/BOF systems developed in this study were manifestations of these challenges: the BOFs were shown to slightly increase the bacterial microbial diversity of the rhizosphere soil, but decreased the bacterial microbial diversity of the bulk soil, and the fungal microbial diversity in both the rhizosphere soil and the bulk soil.

### 4.2. Effect of SynComs/BOFs on Soil Microbial Community and Functions

SynComs can change bacteria and fungi in the cotton rhizosphere soil and enrich beneficial microbes as key species in desertified land [7]. Our study showed that SynComs could change microbial community in soil, but that only a few rare species were enriched, while key species were almost not affected at all, especially key species in the rhizosphere soil, which were stable and under the strong influence of plant roots [9] and hence more resistant to external factors. Core elements of the microbiota, as “hub” microbes, strongly shape the rhizosphere communities [19]. *Sphingomonas* and *Chalara* had were as the key bacteria and fungi in potato rhizosphere soil [15], and they were the core microbes in this study. *Sphingomonas* was a common growth-promoting bacterium found in plants [30]; *Chalara,* on the other hand, was a plant pathogen that mainly presented in the rhizosphere soil [31]. In theory, a reduction in the levels of these would benefit plants. Noticeably, although *Pseudomonas*, *Brevundimonas*, and *Pseudoxanthomonas* were not core bacterial species, they had high abundance in the BOFs, and were hence significantly increased in BOF-treated soils. *Pseudomonas* could promote plant growth and suppress soil-borne pathogens, and was a target genus whose presence was increased in soil by the application of SynComs [25], which were also nitrogen-fixing bacteria [32]. *Brevundimonas* had a *nifH* gene, could colonize in soil around potato roots, and prompt potato growth [33]. *Pseudoxanthomonas* could fix nitrogen and prompt plant growth [34]. Therefore, SynComs that could promote the proliferation of these bacteria in soil would be beneficial for potato plants.

*Plectosphaerella* and *Mortierella* were key fungal genera in FSF and CK, respectively. *Plectosphaerella* were soil saprotrophs, acting as pathogens for some plants, and endophytes for other plants [35]. This genus had biological control agents of potato cyst nematodes [36]. *Mortierella* could prompt plant growth [37]. Its presence was shown in this study to be decreased in organic fertilizer (OF)-treated soil, and conversely increased in BOF-treated soil. *Unclassified_Sordariales*, *Coprinopsis,* and *Fusarium* were not key species, but they had high abundances. The levels of *Sordariales* were enriched in decomposed straw residues [38], and *Coprinopsis* could degrade lignocellulose [39]. *Fusarium* is a genus of pathogenic fungi [15]. In this study, *Coprinopsis* was significantly improved in OF/BOF-treated soil groups, especially in FSB group, while *unclassified_Sordariales* was significantly decreased in OF/BOF-treated soils. *Fusarium* was significantly increased in F and FSF groups, but decreased in FSB and FSBF groups. 

The microbial community determines the microbial functions [21]. *Saprospiraceae* could hydrolyze proteins and degrade complex organic compounds [40], *Moraxellaceae* was shown to be significantly increased during soil hydrocarbon degradation [41], and *Gamma-proteobacteria* were halo-tolerant, contributing to carbon fixation and sulfur oxidation in marine sediments [42]. *Psychrobacter* had extracellular lipolytic activity and carbonic anhydrase genes [43]. *Arenimonas* had genes for acid and alkaline phosphatase, esterase, esterase lipase, lipase and arylamidase [44]. *Luteimonas* had the ability to degrade polycyclic aromatic hydrocarbons [45], and *Oxalobacteraceae* could solubilize phosphate [46]. In this study, BOFs enriched these rare bacteria. In addition, there were significant improvements in *Pseudomonas*, *Brevundimonas,* and *Pseudoxanthomonas*. Thereby, they were shown to be correlated with the improvements in the function categories of Stress_Tolerant, aromatic_compound_degradation, cellulolysis, xylanolyysis, and nitrogen_fixation.

Fungi could colonize plant roots to promote growth, protect roots through constructing relationships with plants, and play important roles in degrading organic materials. Some fungi were opportunistic pathogens, increasing the disease risk for plants [47]. Some species in the *Ceratobasidium* genus were plant pathogens, and some other species in this genus were used to control the presence of *Rhizoctonia solani* in crops [46]. *p_Basidiomycota* and *Ascomycota* had many enzymes that could degrade lignin and plant polysaccharide [48]. *Agaricomycetes* were shown to play important roles in organic compounds breakdown [49]. *Agaricales* could degrade cellulose and lignin [43], *f_Sporormiaceae* could degrade plant residue [50], and many *Lasiosphaeriaceae* species were found on decayed substrates [51]. *C_Pucciniomycetes* includes many important plant pathogens known as rust fungi [47]. In this study, BOFs were shown to enrich these rare fungi. Combined with the core species, they affected fungal functions. Overall, the FSF group was shown to be at increased risk of pathogens such as *Chalara* and *Fusarium*. However, it should be noticed that these pathogens already existed in the soil, and had high abundance. It was interesting that *Fusarium* levels significantly decreased in the FSB and FSBF groups. *Trichoderma*, as a genus of biocontrol fungi, was significantly increased in the bulk soil of FSB and FSBF groups, indicating the interaction and interrelationships between microorganisms, playing important roles in microbial communities, and their functionality. These results also showed the complex nature of the relationships between microorganisms: bacteria–bacteria, bacteria–fungi and fungi–fungi. 

Microbial community and function were also associated with the soil environment [7]. Our results found that BOFs significantly affected soil physicochemical properties, which could influence key bacterial species and core bacteria, promoting potato growth.

## 5. Conclusions

We investigated the colonization of synthetic microbial communities (SynComs), which were selected to cause the potato root exudates associated with continuous cropping obstacles to be removed from bio-organic fertilizers and move into soil. Its effects on potato soil microbial diversity were characterized in this study. The results showed that fungal SynComs were more effective in colonizing both organic fertilizer and soil than bacterial SynComs. BOFs were shown to change soil microbial communities, mainly through activating some rare species, and the abundance of key species and core species, leading to improvements in tolerance of stress, nitrogen fixation, and saprotroph functions. In FSF group, a slight increase in plant pathogens was observed, while in FSB and FSBF groups, the plant pathogens were kept at the same levels as they were F and CK groups. BOFs significantly affected soil physicochemical properties, causing bacterial key species and core bacteria to promote potato growth. In general, the application of SynComs needs to consider the possible elevated risk associated with increased plant pathogens, which will be further studied in our future research.

## Data Availability

The datasets presented in this study can be found in online repositories. The raw data were uploaded into the NCBI Sequence Read Archive (SRA) database (Accession Number: SUB14172939, SUB14370450).

## Supplementary Materials

The following supporting information can be downloaded at: https://www.mdpi.com/article/10.3390/microorganisms12112371/s1, Table S1: Composition and abundance of SCB; Table S2: Composition and abundance of SCF; Table S3: Bacterial abundance in OF and BOF; Table S4: Bacterial abundance in rhizosphere soil and bulk soil; Table S5: Fungal abundance in OF and BOF; Table S6: Fungal abundance in rhizosphere soil and bulk soil.
